# Recent Advances in Long-Acting Drug Delivery and Formulations

**DOI:** 10.3390/pharmaceutics15112519

**Published:** 2023-10-24

**Authors:** Adel Al Fatease, Hamdy Abdelkader

**Affiliations:** Pharmaceutics Department, College of Pharmacy, King Khalid University, Abha 62223, Saudi Arabia; afatease@kku.edu.sa

## 1. Introduction

Conventional immediate-release delivery systems are simple, industrially reproducible, acceptable, and easy-to-use by most patients. Nevertheless, these dosage forms have received critique for not being able to consistently provide optimum therapy for chronic disease conditions, as well as for their potential to induce adverse effects. This is primarily due to the typical rapid, pulse-release and absorption patterns of their drug cargo leading to rapidly fluctuating systemic drug concentration [1].

Long-acting drug delivery systems (LADDS) encompass a range of formulations and technologies that can be used to precisely deliver drug molecules into target tissues. These operate either through systemic circulation or via localized organs/tissues (e.g., skin, eye, and specific lesions) to treat chronic diseases like diabetes, cancer, and brain disorders, as well as for age-related eye diseases. LADDS have been shown to prolong drug release from several hours up to 3 years depending on characteristics of the drug, disease and delivery system [2,3]. LADDS include oral sustained release systems, injectable implants, in situ forming implants, inserts, wafers, transdermal patches, microspheres and nanoparticles [1,4,5]. A number of potential drug classes could be good candidates for LADDS: these include pain killers, biopharmaceuticals, anticancer drugs and centrally acting drugs [2,6].

The Special Issue, entitled “Recent Advances in Long-Acting Drug Delivery and Formulations”, encompasses versatile and innovative research domains of oral, ocular, brain and topical delivery systems and chemical approaches (e.g., prodrugs), highlighting the progress made in identifying excipients (e.g., basic amino acids to ameliorate gastric side effects of non-steroidal anti-inflammatory drugs (NSAIDs)), polymers, and molecular targets to achieve a more effective sustained release systems and safer medicine. A total of 78 authors have contributed to this publication, with 11 original research articles, spanning five contents (Asia, Europe, Africa, North America and Australasia) providing important insights into the pharmaceutical sciences. I would like to encourage the readers to go through each paper and imbibe deeply the published state-of-the-art work in the field; before that, I will to summarize key research findings of interest.

## 2. Overview of the Published Articles

One fascinating paper in this Special Issue concerned the exploration of three basic amino acids (tromethamine, lysine and arginine). These were used to form three salts with ketoprofen (one of commonly prescribed NSAIDs for chronic pain and inflammation). Analgesic activity was significantly enhanced and proceed for these acids in the following order Tris >> lysine > arginine > ketoprofen base. The least severe gastric side effects were experienced by patients treated with lysine and arginine salts.

Abd-Ellah et al. [7] investigated a novel chemical approach (aminoalkoxycarbonyloxymethyl (amino-AOCOM) ether prodrug concept) in order to design a long-acting subcutaneous raltegravir injection for the treatment of HIV.

The corneal permeability in three animal models—rabbit, pigs, and cows— was studied for 25 drugs in an attempt to relate corneal permeability to drug physicochemical properties and tissue thickness via the analysis of quantitative structure permeability relationships (QSPRs) [8]. These twenty-five drugs comprised various drug classes including NSAIDs, β-blockers and corticosteroids commonly prescribed for treating different diseases of the anterior segment of the eye.

One study tested a microwave-treated, physically cross-linked polymer blend film (sodium alginate and carboxymethyl cellulose sodium), prepared via the solvent casting method, for its potential to optimize microwave treatment time. This procedure also allowed researchers to testing for physicochemical attributes and wound healing potential in diabetic animals [9]. The study concluded that the microwave-treated polymer blend films have sufficiently enhanced physical properties, making them an effective candidate for ameliorating the diabetic wound healing process and hastening skin tissue regeneration.

Hybrid gels of hydroxyl propyl methylcellulose (HPMC) and polyvinylpyrrolidone (PVP) were studied in order to enable sustained release topical skin delivery with unique features of superior adhesiveness, spreading ability, and greater cosmetic acceptability than homopolymers alone. These binary hydrogels showed low viscosity, but high bioadhesiveness. Indeed, they still can be applied on the skin comfortably, while providing for the controlled release of the drug over extended periods of time.

Drug repurposing is useful a method for accelerating the drug development process by finding a new clinical use for a substance already on the market and registered for a different indication. On this front, two articles were published that sought to repurpose salbutamol and fenofibrate for treatment of muscle atrophy and retinal diseases, respectively [10,11].

Kumar et al. studied the repurposing potential of the bronchodilator drug salbutamol (β2-receptor agonist) for a beneficial role in diabetics with muscle atrophy. The oral administration of salbutamol increased voluntary muscle strength in diabetic rat models.

In diabetic rats, salbutamol greatly boosted lean muscle mass and grip strength. Additionally, salbutamol therapy increased antioxidant levels in muscles, decreased muscular atrophy and inflammatory markers, and restored muscle damage biomarkers, all of which suggested salbutamol’s potential to lower muscle inflammation and oxidative stress.

Novel 3D polymeric scaffold implantable systems were investigated for intraocular injection in order to retinal diseases. The 3D scaffolds, comprising alginate (ALG) and bovine serum albumin (BSA) containing fenofibrate, were prepared via the freeze-drying technique.

Alzheimer’s disease is a brain ailment that gradually impairs thinking and memory abilities as well as the capacity to perform even the most basic tasks. The majority of Alzheimer’s patients exhibit their initial symptoms later in life. Various estimates show that more than 6 million Americans, the majority of whom are 65 or older, may be affected by Alzheimer’s disease. In this Special Issue, two studies covering novel treatment strategies of Alzheimer’s disease are published. Memantine HCl and Tramiprosate-loaded solid lipid nanoparticles (SLNs) were prepared and characterized for the clearance of Aβ on SHSY5Y cells in a rat hippocampus. Additionally, the neuroprotective effects of cocoa, either alone or in combination with other nutraceuticals, were discussed in the context of an animal model of aluminum-induced Alzheimer’s disease.

In terms of sustained release, forming polymer chitosan has shown superior mechanical properties when cross-linked with trimesic acid. 5-fluorouracil (5-FU)-loaded hydrogels crosslinked with trimesic acid demonstrated the most sustained release profile among the formulations studied in a 3 h period, with 35 to 50% release.

## 3. Limitations and Future Perspectives

In conclusion, LADDS hold promise for the treatment of a variety of chronic diseases, offering safer and more efficient alternatives to the traditional (immediate-release) medication administration method. By creating APIs (small drug molecules or biologics) using long-acting drug delivery systems (LADDS), both efficacy and safety can be improved. LADDS provide advantages that are acknowledged and supported by regulatory bodies, medical professionals, and patients. For individuals who would often need lifelong treatment for debilitating chronic ailments such eye diseases, diabetes, cancer, and brain disorders, LADDS can offer both lasting systemic and local effects.

The cost and accessibility of the biomaterials used to create LADDS, the complexity of some LADDS’ complex systems, and the dependence of some LADDS’ drug carriers on external stimuli (such as light, lasers, and the application of magnetism) to achieve consistent drug release are just several of the hurdles that require resolution. These problems make some LADDS ineligible for regulatory approval. The skyrocketing cost of biomaterials, inadequate scientific knowledge, and a dearth of excipients suitable for recently developing technologies like 3D printing and microneedle systems also hinder the advancement of this technology. The majority of novel long-acting anticancer drug delivery system concepts have been tested in animal models; nevertheless, regulatory organizations have voiced strong ethical and technological objections to translating such success in animal models to clinical phases.

Finally, the guest editors of this Special Issue extend their sincere appreciation for all the authors and reviewers who responded to our invitation. Your willingness to share the outcomes of your outstanding research has greatly enriched this collection, and your contributions have been invaluable in the meticulous evaluation of the manuscripts.
